# From Self-efficacy to Imposter Syndrome: The Intrapersonal Traits of Surgeons

**DOI:** 10.5435/JAAOSGlobal-D-22-00051

**Published:** 2022-04-12

**Authors:** Alexandra Medline, Helyn Grissom, Ndéye F. Guissé, Victoria Kravets, Sandra Hobson, Julie Balch Samora, Mara Schenker

**Affiliations:** From the Emory University School of Medicine, Atlanta, GA (Medline, Dr. Grissom, Kravets, Dr. Hobson, and Dr. Schenker); the Emory University Department of Orthopaedic Surgery, Atlanta, GA; the Washington University Department of Orthopaedic Surgery, St. Louis, MO (Dr. Guissé); and the Nationwide Children's Hospital, Columbus, OH (Dr. Samora).

## Abstract

**Background:**

Demographic differences among surgical trainees regarding intrapersonal traits, such as imposter syndrome and assertiveness, have become widely acknowledged. However, many of these characteristics have not been examined in tandem, nor among both trainees and surgeons in practice. This study aimed to address these knowledge gaps.

**Methods:**

This was an anonymous, voluntary survey study comprised of validated measures of (1) self-efficacy, (2) imposter syndrome, (3) assertiveness, (4) perfectionism, and (5) self-rated likeability. A multimodal recruitment strategy was used and surgeons across all subspecialties were eligible for inclusion.

**Results:**

A total of 296 participants were included, with 54% identifying as female (n = 161) and 72% between the ages of 25 and 40 years of age (n = 212). Imposter syndrome, assertiveness, and perfectionism scales were normally distributed; self-efficacy and self-rated likeability scales demonstrated slight negative skew. Self-identified male sex was associated with less imposter syndrome (*P* < 0.001) and perfectionism (*P* = 0.035) and higher assertiveness (*P* < 0.001). Imposter syndrome was less common among older age groups (*P* = 0.001).

**Conclusions:**

Surgeons are a self-efficacious group of perfectionists with widespread variability in imposter syndrome and assertiveness. Female sex and younger age were associated with more imposter syndrome and less assertiveness, highlighting an opportunity for early career coaching.

Building a successful and long-standing career in surgery—from residency through practice—is contingent on factors beyond technical prowess. A wide range of personal traits, although seemingly more difficult to quantify than technical competency alone, have become increasingly recognized as pivotal to physician mental health, well being and in preventing physician burnout.^1^ By contrast, traits such as maladaptive perfectionism and imposter syndrome have been shown to be associated with increased rates of negative mental health effects^1,2^ and impaired job performance.^3,4^ Given the pervasiveness and negative sequelae of burnout,^5-7^ in addition to generalized career dissatisfaction in surgery,^8^ there is an urgent need to evaluate traits distinct from surgical skill, such as self-efficacy, imposter syndrome, assertiveness, and perceived likeability.

It has been widely established that there are gender differences among these traits. For example, female surgical residents report a relative lack of both self-perceived clinical confidence and autonomy in comparison with their equally qualified male peers. For other traits such as self-efficacy, the effects of sex are mixed or have yet to be analyzed among surgical trainees.^9^ Self-rated likeability is another trait that has not been thoroughly analyzed among this population and was chosen as our fifth trait of interest, given that it has been shown to correlate with maladaptive personality traits,^10^ self-esteem,^11^ and individual differences in anxiety, depression, and avoidance.^12^ Although the potential gender differences among these traits have been increasingly emphasized, no study to the best of our knowledge has yet to evaluate a broad combination of these characteristics among both surgical trainees and practicing surgeons.

This study aimed to (1) assess the prevalence and scale of five intrapersonal traits among surgical residents and practicing surgeons and (2) identify potential associations between the presence of these traits and demographic factors such as self-identified sex, age, and time in practice.

## Methods

This was an International Review Board-approved study consisting of an anonymous and voluntary RedCAP survey including validated measures of (1) self-efficacy (New General Self-Efficacy Scale,^13^ score range 8 to 40), (2) imposter syndrome (Clance Imposter Phenomenon Scale,^14^ score range 20 to 100), (3) assertiveness (Rathus Assertiveness Scale–Short Form,^15^ score range −57 to 57), and (4) perfectionism (Short Almost Perfect Scale,^16^ score range 8 to 56). A Likert scale (ranging from 1 to 7) was used to measure self-rated likeability; this method has been used by several peer-reviewed studies that have evaluated this trait.^10,17,18^ Among all five traits, a higher score on any individual assessment signified a higher degree of the characteristic. The individual assessments were shown without a title or label, and thus, respondents were not made aware of which traits were being analyzed until the completion of the survey. This was done to prevent respondents from altering their responses based on their own perception or prejudice of the topic in question.

Current residents, fellows, and attending physicians among all surgical subspecialties were eligible for participation. A multimodal recruitment strategy using social media, e-mail, and other internet-mediated methods was used to recruit eligible participants.^19^ Demographics collected included age, self-identified sex, race, surgical subspecialty, and training level. Region of practice was also collected and categorized as (1) midwest, (2) northeast, (3) south, or (4) west. On completion, respondents were provided with their scores for each individual assessment and a detailed explanation of score interpretation.

Mann-Whitney U tests, Student *t*-tests, Analysis of Variance (ANOVA), Tukey's Honest Significant Difference (HSD) test, Spearman correlation analyses, and linear regression models were conducted to examine associations within the collected data. Kolmogorov-Smirnov testing and histogram analysis were used for normality testing, and any variables not normally distributed were analyzed with nonparametric tests.

## Results

Two hundred ninety-six respondents were included, with 168 (56.8%) who completed the survey in its entirety. A slight majority of respondents (n = 161, 54.4%) identified as female, while the most common age group (28.7%) was between 30 and 34 years (Table 1). One hundred sixty-five participants (55.7%) were attending surgeons, with the remaining participants in various stages of surgical training. No significant differences were observed in any of the five traits when comparing surgeons in practice and those in training (*P* > 0.05).

**Table 1:** Study Participant Characteristics (N = 296), 2020 to 2021

Variable Response	N (%)
Age (in yrs)	
25-29	63 (21.4)
30-34	85 (28.7)
35-39	64 (21.6)
40-44	27 (9.1)
45-49	25 (8.4)
50-59	21 (7.1)
60+	10 (3.4)
Sex	
Female	161 (54.4)
Male	132 (44.6)
Race	
White	236 (79.7)
Asian/Pacific Islander	42 (14.2)
Hispanic or Latino	15 (5.1)
Black or African American	12 (4.1)
Other	6 (2.0)
Current level of training	
Attending physician	165 (55.7)
Fellow	12 (4.1)
PGY-1	31 (10.5)
PGY-2	27 (9.1)
PGY-3	27 (9.1)
PGY-4	21 (7.1)
PGY-5/6	24 (8.1)
Current region of practice	
Midwest	43 (14.5)
Northeast	36 (12.2)
South	136 (45.9)
West	80 (27.0)
Current specialty	
Orthopaedics	245 (82.8)
Obstetrics and Gynecology	11 (3.7)
Urology	10 (3.4)
General surgery	9 (3.0)
Colorectal surgery	3 (1)
Gynecologic oncology	2 (0.7)
Thoracic surgery	1 (0.3)
Ophthalmology	1 (0.3)
Otolaryngology	1 (0.3)
Other	14 (4.7)

**Figure 1:**
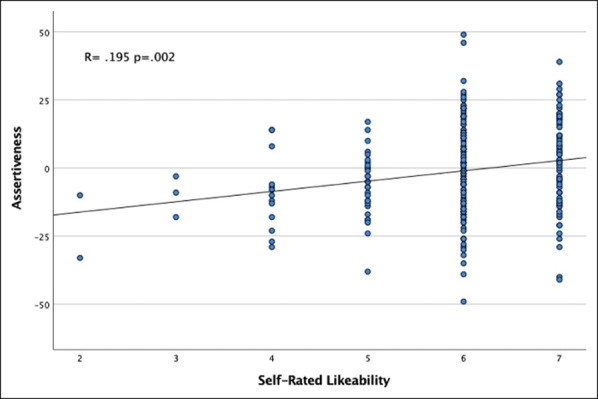
Graph showing correlation between assertiveness and self-rated likeability

Among all participants, the mean self-efficacy score was 34.2 ± 4.4, indicating a highly self-efficacious group. The imposter syndrome and assertiveness scales were normally distributed with a mean of 60.5 ± 16.5 and −1.3 ± 17.0, respectively. Perfectionism was also normally distributed with a mean of 43.2 ± 7.2, indicating an overall trend toward perfectionism. The mean self-rated likeability was high at 6.0 (range 2.0 to 7.0; skew −1.35). Including all sex and age groups, those who reported themselves as likeable were found to score higher on the assertiveness scale (R = 0.195, *P* = 0.002; Figure 1).

Compared with female surgeons, male surgeons exhibited less imposter syndrome (*P* < 0.001) and perfectionist traits (*P* = 0.035), but more assertiveness (*P* < 0.001). No difference between men and women was observed in measures of self-efficacy (*P* = 0.162) or self-rated likeability (*P* = 0.153).

When comparing age groups, the only significant difference detected was for imposter syndrome, which was found to be less common in older age (*P* ≤ 0.001). Post hoc analysis revealed that significant differences were between the age group of 30 to 34 years compared with 45 to 49 years (*P* = 0.040) and 50 to 59 years (*P* < 0.001).

For linear regression analysis (Supplemental Table 1, http://links.lww.com/JG9/A213), no demographic variables of interest, including age, sex, race, time, or region of practice, were associated with increased self-efficacy. Female sex was predictive of higher levels of imposter syndrome (*P* = 0.002) and lower levels of assertiveness (*P* < 0.001). When controlling for age, race, and sex, practicing in the northeast was the only predictive variable of increased levels of perfectionism (*P* = 0.014). Practicing in the midwest was negatively predictive of self-rated likeability (*P* = 0.002), whereas self-identifying as Black/African American was positively predictive of self-rated likeability (*P* = 0.023).

## Discussion

Our results indicate that surgeons are, across all demographic groups, a highly self-efficacious group of perfectionists, with significant demographic variation illustrated among imposter syndrome and assertiveness. Notably, our study demonstrates these effects among both trainees and surgeons in practice. Furthermore, in the context of the wide range of studies that have evaluated demographic variation in these traits, a notable and unique strength of our study is that validated scores of four different characteristics were evaluated concomitantly, in addition to self-rated likeability. This allowed us to assess demographic associations and how these characteristics interrelate.

The finding that women reported more imposter syndrome and perfectionist tendencies and less assertiveness coincides with previous literature demonstrating gender differences in self-evaluation and confidence among surgical residents.^20-22^ Previous literature has also suggested that for women, assertiveness and confidence may be inversely related to likeability, and the desire to be “liked” may cause women to act and behave more timidly.^20,23^ Be that as it may, in our study, there was a markedly positive association between assertiveness and likeability, which suggests that those who believe that they are admired by others may feel more comfortable acting assertive. Assertiveness has a direct effect on many facets of life in surgery, extending from surgical education (ie, willingness to take the knife) to leadership growth and development. It is important that future research continues to elucidate the association between likeability and assertiveness, especially among women.

Despite the recognition of gender differences among surgical trainees, a knowledge gap remains surrounding potential interventions to reduce these discrepancies. Although interventions have been evaluated among nonsurgical populations, such as benefits of assertiveness workshops among nursing and high-school students and one study among medical students,^24-26^ the effects among surgeons and other medical professions are still largely unknown. Given that multiple previous studies have shown that female surgeons experience higher degrees of burnout compared with their male counterparts,^27,28,29,30^ it is imperative this gender gap is addressed. Although standardizing operative and clinical experiences may help develop confidence in all surgical trainees,^21^ our findings suggest that more awareness should be given younger female trainees to identify who may benefit from supplemental mentorship, both within and outside of the operating room.

Professional development programs and strong directed mentorships that allow for the expression of personal and professional concerns in a safe environment may prove to be beneficial.^28^ Mentorship, in addition to age, motherhood, and more advanced academic status, has been shown to be associated with higher levels of confidence among female plastic surgeons.^31^ Furthermore, as recently described by Babchenko et al, mastery of surgical skill may be described as similar to becoming an elite athlete, and thus, female surgeons could benefit from lessons learned from optimal coaching in sports.^20^ In addition, implicit gender bias has also been identified as a key contributor to gender discrepancies. Methods to combat these biases include entrusting women with more responsibility and key operational steps earlier and more often in their training.^20^

Imposter syndrome represents a maladaptive thought process leading one to believe that their success has been solely due to luck and to fear that others may discover they are neither intelligent nor capable.^32^ In addition to sex, younger age and earlier years of training were found to be associated with imposter syndrome, but unlike sex, were not found to be associated with our other traits of interest. This finding demonstrates the importance of early interventions targeted at this phenomenon. In our study, a question that demonstrated particularly high gender discrepancy related to imposter syndrome was, “*I avoid evaluations if possible and have a dread of others evaluating me*.” The concerning reality that younger and female trainees avoid evaluation and the potential opportunity for growth highlights again the pivotal role of mentorship and, more specifically, of the early identification of imposter syndrome and other related traits, consistent with underdeveloped confidence.

One limitation of our study was the potential for nonresponse bias due to the use of an online survey method. In addition, no information regarding potential personality or psychiatric conditions was collected, which may also have influenced our results or introduced response bias. A third limitation was due to our chosen recruitment method because the survey was distributed widely on the internet, rendering no tracking of the total number of individuals contacted or the overall response rate. Yet another limitation is that most of our respondents were within orthopaedic surgery by virtue of the fact that the majority of the authors practice orthopaedics and were thus more easily able to recruit colleagues as respondents. Additional studies may look further to understand the relationship between surgical subspecialties and these important intrapersonal traits. Given that our study found that practicing in the northeast was predictive of increased levels of perfectionism, additional studies may also delve further into regional differences between these intrapersonal traits. This regional finding may indicate that surgical training programs clustered in the northeast are more selective for applicants that communicate higher levels of perfectionism, or it may be that these programs engender this trait during the received training. Similarly, additional studies may also continue to evaluate the association between race and these intrapersonal traits. Our study found no racial differences for each of our traits of interest except self-rated likeability, for which Black or African American race was found to be positively predictive and could warrant additional qualitative analysis. Above all, there is a clear crisis of underrepresentation of surgical trainees of color, and especially women in color, across the country, and thus an imminent need to ensure representation, sponsorship, and the cultivation of an inclusive environment across all levels of surgical training.^33^

In conclusion, the results of this survey highlight the widespread variability in imposter syndrome and assertiveness among surgical trainees and surgeons, with markedly more imposter syndrome and less assertiveness among female trainees. Given that intrapersonal traits such as these have now been consistently validated as quantitatively measurable, there is a need for the systematic evaluation of these traits in the same fashion as other skills that have been routinely emphasized in the field, such as surgical technique. Nevertheless, we appreciate that variation in these intrapersonal traits exist between individuals to an even larger degree then between sexes, age groups, and regions. We thus envision a personalized educational reform that is informed by the gender and age discrepancies illustrated in our study, whereby trainees could a priori identify individualized traits in which they have lower scores.^20^ We will not be able to address variation in these traits without starting to routinely measure them. With appropriate resources, students, and women in particular, could be empowered to take accountability for their own personal growth, fostering a more nurturing and proactive surgical culture.^34^

## Supplementary Material

SUPPLEMENTARY MATERIAL

## References

[1] Thomas M Bigatti S: Perfectionism, impostor phenomenon, and mental health in medicine: A literature review. Int J Med Educ 2020;11:201-213. 32996466 10.5116/ijme.5f54.c8f8 PMC7882132

[2] Legassie J Zibrowski EM Goldszmidt MA: Measuring resident well-being: Impostorism and burnout syndrome in residency. J Gen Intern Med 2008;23:1090-1094. 18612750 10.1007/s11606-008-0536-x PMC2517942

[3] Leach PK Nygaard RM Chipman JG Brunsvold ME Marek AP: Impostor phenomenon and burnout in general surgeons and general surgery residents. J Surg Educ 2019;76:99-106. 30122638 10.1016/j.jsurg.2018.06.025

[4] Villwock JA Sobin LB Koester LA Harris TM: Impostor syndrome and burnout among American medical students: A pilot study. Int J Med Educ 2016;7:364-369. 27802178 10.5116/ijme.5801.eac4 PMC5116369

[5] Balch CM Freischlag JA Shanafelt TD: Stress and burnout among surgeons: Understanding and managing the syndrome and avoiding the adverse consequences. Arch Surg 2009;144:371-376. 19380652 10.1001/archsurg.2008.575

[6] Patel RS Bachu R Adikey A Malik M Shah M: Factors related to physician burnout and its consequences: A review. Behav Sci (Basel) 2018;8:98. 30366419 10.3390/bs8110098 PMC6262585

[7] Shanafelt TD Mungo M Schmitgen J : Longitudinal study evaluating the association between physician burnout and changes in professional work effort. Mayo Clin Proc 2016;91:422-431. 27046522 10.1016/j.mayocp.2016.02.001

[8] Balch CM Shanafelt TD Sloan JA Satele DV Freischlag JA: Distress and career satisfaction among 14 surgical specialties, comparing academic and private practice settings. Ann Surg 2011;254:558-568. 21946217 10.1097/SLA.0b013e318230097e

[9] Milam LA Cohen GL Mueller C Salles A: The relationship between self-efficacy and well-being among surgical residents. J Surg Educ 2019;76:321-328. 30245061 10.1016/j.jsurg.2018.07.028 PMC6380924

[10] Lamkin J Maples-Keller JL Miller JD: How likable are personality disorder and general personality traits to those who possess them? J Pers 2018;86:173-185. 28124372 10.1111/jopy.12302

[11] Brockner J Lloyd K: Self-esteem and likability: Separating fact from fantasy. J Res Personal 1986;20:496-508.

[12] Klein AM Houtkamp EO Salemink E Baartmans JMD Rinck M van der Molen MJ: Differences between self- and peer-rated likability in relation to social anxiety and depression in adolescents with mild intellectual disabilities. Res Dev Disabil 2018;80:44-51. 29908392 10.1016/j.ridd.2018.05.016

[13] Chen G Gully SM Eden D: Validation of a new general self-efficacy scale. Organizational Res Methods 2001;4:62-83.

[14] Clance PR: Clance's IP Scale: The impostor phenomenon: When success makes you feel like a fake. Toronto, Ontario, Canada: Bantam Books, 1986.

[15] Jenerette C Dixon J: Developing a short form of the simple Rathus assertiveness schedule using a sample of adults with sickle cell disease. J Transcult Nurs 2010;21:314-324. 20592057 10.1177/1043659609360712

[16] Rice KG Richardson CM Tueller S: The short form of the revised almost perfect scale. J Pers Assess 2014;96:368-379. 24090303 10.1080/00223891.2013.838172

[17] Sullivan GM Artino AR Jr: Analyzing and interpreting data from Likert-type scales. J Grad Med Educ 2013;5:541-542. 24454995 10.4300/JGME-5-4-18 PMC3886444

[18] Swann WB Jr: Self-verification processes: How we sustain our self-conceptions. J ofExperimental Soc Psychol 1981;17:351-372.

[19] McRobert CJ Hill JC Smale T Hay EM van der Windt DA: A multi-modal recruitment strategy using social media and internet-mediated methods to recruit a multidisciplinary, international sample of clinicians to an online research study. PLoS One 2018;13:e0200184. 29979769 10.1371/journal.pone.0200184 PMC6034855

[20] Babchenko O Gast K: Should we train female and male residents slightly differently? JAMA Surg 2020;155:373-374. 32129814 10.1001/jamasurg.2019.5887

[21] Bucholz EM Sue GR Yeo H Roman SA Bell RH Jr Sosa JA: Our trainees' confidence: Results from a national survey of 4136 US general surgery residents. Arch Surg 2011;146:907-914. 21844434 10.1001/archsurg.2011.178

[22] Flyckt RL White EE Goodman LR Mohr C Dutta S Zanotti KM: The use of laparoscopy simulation to explore gender differences in resident surgical confidence. Obstet Gynecol Int 2017;2017:1945801. 28203253 10.1155/2017/1945801 PMC5288545

[23] Shipman KKC The confidence gap. 2014.

[24] Ayhan D Seki Öz H: Effect of assertiveness training on the nursing students' assertiveness and self-esteem levels: Application of hybrid education in COVID-19 pandemic. Nurs Forum 2021. 10.1111/nuf.12610 PMC8242798 34028042

[25] Eslami AA Rabiei L Afzali SM Hamidizadeh S Masoudi R: The effectiveness of assertiveness training on the levels of stress, anxiety, and depression of high school students. Iran Red Crescent Med J 2016;18:e21096. 26889390 10.5812/ircmj.21096 PMC4752719

[26] Lin Y-R Shiah IS Chang YC Lai TJ Wang KY Chou KR: Evaluation of an assertiveness training program on nursing and medical students' assertiveness, self-esteem, and interpersonal communication satisfaction. Nurse Educ Today 2004;24:656-665. 15519449 10.1016/j.nedt.2004.09.004

[27] Dimou FM Eckelbarger D Riall TS: Surgeon burnout: A systematic review. J Am Coll Surg 2016;222:1230-1239. 27106639 10.1016/j.jamcollsurg.2016.03.022 PMC4884544

[28] Elmore LC Jeffe DB Jin L Awad MM Turnbull IR: National survey of burnout among US general surgery residents. J Am Coll Surg 2016;223:440-451. 27238875 10.1016/j.jamcollsurg.2016.05.014 PMC5476455

[29] Galaiya R Kinross J Arulampalam T: Factors associated with burnout syndrome in surgeons: A systematic review. Ann R Coll Surg Engl 2020;102:401-407. 32326734 10.1308/rcsann.2020.0040 PMC7388944

[30] Linzer M Harwood E: Gendered expectations: Do they contribute to high burnout among female physicians? J Gen Intern Med 2018;33:963-965. 29435727 10.1007/s11606-018-4330-0 PMC5975148

[31] Van Boerum MS Jarman AF Veith J : The confidence gap: Findings for women in plastic surgery. Am J Surg 2020;220:1351-1357. 32746978 10.1016/j.amjsurg.2020.06.037

[32] Feenstra S Begeny CT Ryan MK Rink FA Stoker JI Jordan J: Contextualizing the impostor syndrome. Front Psychol 2020;11:575024. 33312149 10.3389/fpsyg.2020.575024 PMC7703426

[33] Ellis DI Khubchandani JA: The state of diversity in American surgery: A call to action. Ann Surg 2021;273:e3-e4. 33065639 10.1097/SLA.0000000000004518 PMC7737864

[34] Ward S Outram S: Medicine: In need of culture change. Intern Med J 2016;46:112-116. 26813903 10.1111/imj.12954

