# Supplementary material for: From Self-efficacy to Imposter Syndrome: The Intrapersonal Traits of Surgeons
Source: J Am Acad Orthop Surg Glob Res Rev. 2022 Apr 12;6(4):e22.00051. doi: 10.5435/JAAOSGlobal-D-22-00051 (PMC10566864; doi:10.5435/JAAOSGlobal-D-22-00051)
Supplement: SUPPLEMENTARY MATERIAL [file jagrr-6-e22.00051-s001.docx]

***Supplemental Table 1: Linear Regression Models Estimating Effects of Demographic Variables on 5 Intangible Traits (N =296)***

| **Exposure Variables** | **Self-Efficacy** | | **Imposter Syndrome** | | | **Assertiveness** | | | **Perfectionism** | | **Self-rated Likeability** | |
| --- | --- | --- | --- | --- | --- | --- | --- | --- | --- | --- | --- | --- |
|  | **β** | **Sig.** | **β** | **Sig.** | | **β** | **Sig.** | | **β** | **Sig.** | **β** | **Sig.** |
| **Region of practice*** |  | | | | | | | | | | | |
| Midwest | -0.83 | .247 | 0.10 | .151 | .017 | | | .812 | .015 | .845 | -.227 | **.002***** |
| Northeast | -1.09 | .116 | 0.17 | .011 | | -.089 | | .203 | .180 | **.014**** | -.124 | .087 |
| West | -0.02 | .731 | -0.01 | .871 | | .020 | | .773 | -.062 | .400 | .017 | .818 |
| **Age** | -0.65 | .467 | -0.17 | **.049**** | | .089 | | .325 | -.166 | .079 | -.055 | .553 |
| **Gender** |  | | | | | | | | | | | |
| Female | -0.06 | .379 | 0.22 | **.002***** | | -.326 | | **<.001***** | .103 | .173 | -.039 | .600 |
| **Current Level of Training** | 0.12 | .200 | -0.05 | .615 | | -.009 | | .923 | .010 | .920 | -.005 | .956 |
| **Race/ Ethnicity** |  | | | | | | | | | | | |
| White | 0.01 | .935 | -0.09 | .371 | | .065 | | .524 | .009 | .932 | .070 | .517 |
| Hispanic/ Latino | .063 | .383 | -.031 | .658 | | -.005 | | .949 | **.002***** | .983 | .081 | .283 |
| Black/ African American | .020 | .781 | -.058 | .406 | | .080 | | .260 | -.491 | .624 | .168 | **.023**** |
| Asian/ Pacific Islander | -.093 | .347 | .017 | .860 | | -.005 | | .958 | .182 | .855 | .071 | .485 |

**South region coded as dummy variable*

**p < .05

***p<.01
